# Transactivation of Human Endogenous Retroviruses by Viruses

**DOI:** 10.3390/v16111649

**Published:** 2024-10-22

**Authors:** Erin F. Evans, Ananya Saraph, Maria Tokuyama

**Affiliations:** Department of Microbiology and Immunology, Life Sciences Institute, The University of British Columbia, Vancouver, BC V6T 1Z4, Canada

**Keywords:** endogenous retroviruses, viral infection, transactivation

## Abstract

Human endogenous retroviruses (HERVs) are remnants of ancient retroviral infections that are part the human genome and are normally silenced through epigenetic mechanisms. However, HERVs can be induced by various host and environmental factors, including viral infection, and transcriptionally active HERVs have been implicated in various physiological processes. In this review, we summarize mounting evidence of transactivation of HERVs by a wide range of DNA and RNA viruses. Though a mechanistic understanding of this phenomenon and the biological implications are still largely missing, the link between exogenous and endogenous viruses is intriguing. Considering the increasing recognition of the role of viral infections in disease, understanding these interactions provides novel insights into human health.

## 1. Introduction

Endogenous retroviruses (ERVs) are viral sequences that are part of eukaryotic genomes including the human genome [1]. ERVs are remnants of ancient retroviral infection and genomic integration events that date as far back as eighty million years ago [2,3]. Viral sequences that integrated into the germline and became fixed through generations now make up 8% of the human genome [4,5,6]. An integrated retroviral genome is called a provirus and consists of 5′ and 3′ long terminal repeats (LTRs) flanking viral group-specific antigen (*gag*), polymerase (*pol*) and envelope (*env*) genes. Human ERVs (HERVs) are proviral sequences that can produce RNA and viral proteins but are no longer replication-competent due to inactivating mutations [7]. HERVs are associated with inflammation in a range of diseases including autoimmunity, neurodegenerative diseases and cancer [8,9,10,11,12]. Moreover, HERVs are commonly elevated during viral infection and may have detrimental or beneficial effects [13,14,15]. Considering the increasing incidence of infectious diseases around the world [16] and association of viral infections with autoimmune disease [17,18,19], HERV activation by viruses is a phenomenon that is worthwhile understanding in more depth.

There are more than 500,000 copies of ERV sequences in the human genome, and they are divided into three classes: Class I (gammaretrovirus-like), Class II (betaretrovirus-like) and Class III (spumaretrovirus-like) [20]. HERV-H and -W (Class I) and HERV-K (Class II) are the most studied in the context of viral infection and are discussed most in this review. HERV-H is the oldest, most abundant HERV, and many copies retain *gag,* though not *env* [21,22,23]. HERV-W is best known for Syncytin-1 (Syn-1), an HERV-W envelope protein that has been co-opted to mediate the fusion of trophoblast cells during placental development [24,25,26]. HERV-K elements called human mouse mammary tumor virus like 1–10 (HML-1–HML-10) are the youngest HERVs that were actively replicating as recently as 250,000 years ago and thus have the most intact proviral sequences [27,28,29]. Some HERV-K elements remain unfixed and are insertionally polymorphic within the human population [30,31,32]. Other HERVs, such as ERV-3, ERV-9 and ERV-E, are not well studied in the context of viral infection. Although HERV families are often described at the family level, it is worth noting that each one of these families represents hundreds of genomic copies, and we are far from understanding the physiological relevance of HERVs at the level of individual loci.

Approximately 30% of HERVs are transcriptionally active in a wide range of normal tissues and cell types [33,34,35]. ERVs are regulated epigenetically and transcriptionally [36,37,38]. Kruppel-associated box zinc finger proteins (KRAB-ZFPs) repress ERV expression by directly binding to the DNA and recruiting tripartite motif-containing 28 (TRIM28) and histone methyltransferase SETDB1, which adds H3K9 histone trimethylation (H3K9me3) marks [39,40]. Chromatin remodeling during viral infection can derepress ERVs through opening of the chromatin resulting in aberrant expression of ERVs. The first such report in 1993 showed that herpes simplex virus 1 (HSV-1) infection causes reactivation of multiple sclerosis-associated retrovirus (MSRV), later classified as HERV-W, in leptomeningeal cells (LM7) [41]. Many reports following this initial study have shown expression of HERVs during viral infection or transactivation of HERVs by viral proteins. How these changes in ERV expression affect inflammatory outcomes during infection or chronic disease is not known.

**Figure 1:**
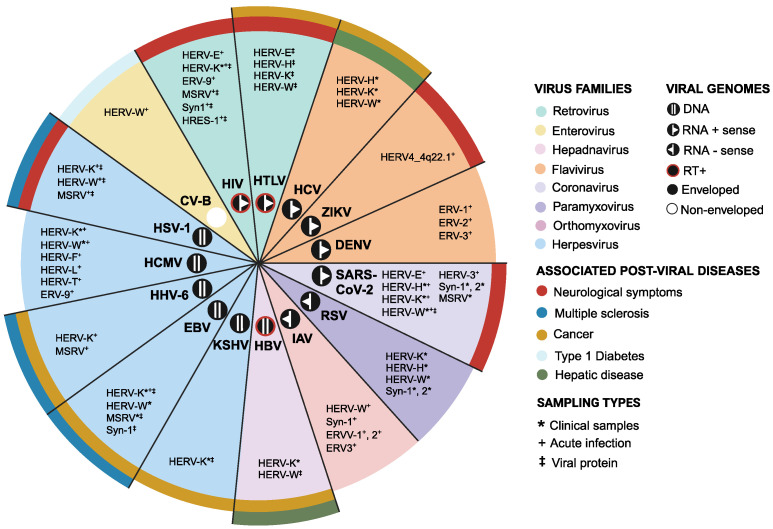
Summary of HERV activation by viruses. HERVs that are activated by the indicated viruses and virus families are listed along with the types of samples where HERVs are detected. The post-viral diseases that are associated with viruses are also highlighted.

Studies of HERVs have greatly benefited from advancements in detection methods and improvements in annotations of HERVs. There is now a wealth of studies that have reported on the dynamic expression of HERVs in various cell types and conditions during viral infection (Supplementary Table S1). In this review, we survey published works that have shown elevated HERV expression during infection by a range of DNA and RNA viruses and categorize them by virus and HERV family. Further, we begin to explore links between HERV transactivation and viral infections that are strongly associated with diseases such as multiple sclerosis (MS) and type 1 diabetes (Figure 1). Collectively, these studies reveal an interesting crosstalk between endogenous and exogenous viruses, warranting further investigation into these links and their potential contribution to health and disease.

## 2. DNA Viruses

### 2.1. Herpes Simplex Virus 1 (HSV-1)

HSV-1 is an alphaherpesvirus that is transmitted through the oral or genital route, causing oral and genital herpes. Approximately 67% of the global population under the age of 50 is infected with HSV-1 [42]. After an initial lytic infection of epithelial cells, HSV-1 establishes latency in sensory neurons and remains in the host for life. Periodic reactivation of latent HSV-1 is associated with recurrent clinical symptoms [43]. Some recent studies have also proposed a link between HSV-1 infection and the development of MS [44,45].

**Figure 2:**
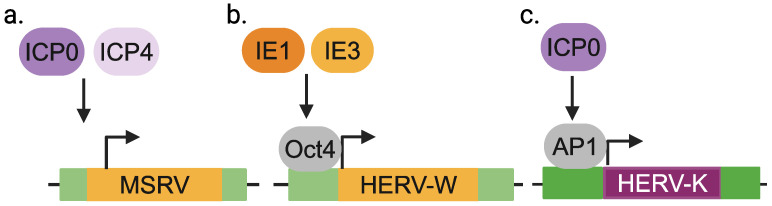
HERV activation by HSV-1. Viral proteins and host transcription factors that are involved in the activation of MSRV (**a**), HERV-W (**b**) and HERV-K (**c**) in context of HSV-1 infection.

HERV-W and K: MSRV virions that belong to the HERV-W family were originally isolated from LM7 cells derived from an MS patient [46] and were shown to be amplified upon HSV-1 infection [41]. MSRV production does not seem to require viral replication, as the HSV-1 immediate early (IE) proteins, ICP0 and ICP4, are sufficient to amplify MSRV (Figure 2a). Since this initial finding, other studies have shown similar effects of HSV-1 on HERVs. For instance, HERV-W transcripts are elevated in HeLa cells infected with HSV-1 [47]. Overexpression of the IE genes IE1 or IE3 is sufficient for HERV-W induction, which depends on Oct1 transcription factor binding to the HERV-W LTR (Figure 2b). HSV-1 upregulates HERV-W *env* and *gag* in human neuroblastoma cells (SK-N-MC and IMR-32) [48], human brain microvascular endothelial cells (HBMEC) and primary human cerebral endothelial cells [49]. It has also been shown that UV-inactivated HSV-1 enhances reverse transcriptase (RT) activity in PBMCs from MS patients compared to PBMCs from healthy donors, though RT activity may originate from sources other than HERVs [50]. Finally, HERV-K transcripts are elevated in human teratocarcinoma (Tera-2) cells infected with HSV-1 or expressing HSV-1 IE protein ICP0, and this induction requires the Ap1 transcription factor binding site within the HERV-K LTR (Figure 2c) [51], suggesting a role for AP1 in the expression.

### 2.2. Human Cytomegalovirus (HCMV)

An estimated 80% of adults around the world are infected with HCMV [52]. HCMV is a betaherpesvirus that is most commonly acquired during childhood and is transmitted through bodily fluids. HCMV is capable of infecting a broad range of cell types and establishes latency in the bone marrow hematopoietic progenitor cells [53]. In immunocompromised hosts, HCMV can reactivate, replicate to high levels and cause end organ disease [54].

HERV-W and K: HCMV infection was first shown to activate HERV-K expression in human embryonic lung fibroblasts [55]. HERV-K and HERV-W *pol* are also elevated in the sera of kidney transplant patients with high levels of HCMV infection [56]. In GliNS1 neural tumor cells and human umbilical vein endothelial cells (HUVECs), HCMV infection upregulates class I (HERV-T, HERV-W, HERV-F, ERV-9), class II (HERV-K) and class III (HERV-L) retroelements [57]. HERV-K (HML-2) transcription is reduced when infected cells are treated with ganciclovir, a viral DNA replication inhibitor, but unaffected by siRNA-mediated silencing of IE viral genes, indicating that early and late viral gene products are likely required. HCMV is known to cause hypomethylation of the host DNA by regulating the expression of DNA methyltransferase 1 (DNMT1) and 3 (DNMT3) [58]. Given the role of DNA methylation in ERV silencing, derepression of epigenetic silencing of HERVs may be a possible mechanism of HERV expression during HCMV infection.

### 2.3. Human Herpesvirus 6 (HHV-6)

HHV-6 is a betaherpesvirus that preferentially replicates in activated CD4+ T cells and establishes latency in bone marrow progenitor cells [59] and in the central nervous system [60]. HHV-6 is usually acquired early in childhood [61,62] and is associated with febrile diseases [62]. HHV-6 is also implicated in chronic diseases and malignancies such as MS [63], Hashimoto’s thyroiditis [64] and Hodgkin’s disease [65].

HERV-W and K: Upon HHV-6A infection, MSRV *env* as well as MSRV env and gag proteins are increased in T lymphoblasts (HSB-2), primary cord blood mononuclear cells and glioblastoma cells (U-87 MG). MSRV *env* induction occurs through the HHV-6A receptor, CD46-Cyt1 isoform [66] and is dependent on protein kinase C (PKC) signaling [67]. HERV-K18 *env* is increased upon HHV-6A infection of HSB2-ML and HSB-2 cells [68] and HHV-6B infection of PBMCs [69]. Blocking HHV-6B glycoprotein H (gH), the CD46 receptor, or treatment of infected cells with cycloheximide decreases HERV-K18 *env* expression, while viral DNA replication and expression of late genes are not necessary for HERV-K18 activation [69]. This indicates that HERV-K18 activation requires viral attachment and de novo protein synthesis during HHV-6B infection.

### 2.4. Epstein–Barr Virus (EBV)

EBV is a gammaherpesvirus and a common childhood infection that has a seroprevalence rate of approximately 95% in the adult population. EBV infection is usually asymptomatic, but infection during late childhood or early adulthood can cause infectious mononucleosis (IM). EBV infects epithelial cells and B cells and establishes latency in memory B cells [70,71]. EBV has oncogenic properties and is estimated to contribute to 265,000 cases of cancer globally [72]. EBV has also been long been linked to MS [73]. Recently, a longitudinal analysis of 10 million adults showed a 32-fold increase in the risk of developing MS following infection with EBV [74], particularly linked to individuals with IM [75]. Incidentally, HERVs are activated by EBV and have been implicated in MS.

**Figure 3:**
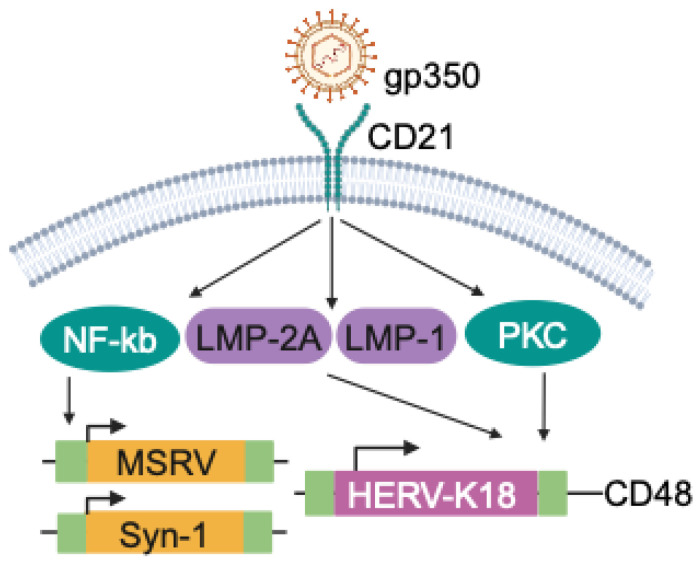
Activation of HERVs by EBV. Expression of MSRV, Syn-1 and HERV-K18 are regulated by viral and host factors that are expressed or activated during EBV infection.

HERV-W: EBV glycoprotein 350 (gp350) is expressed on the viral envelope and binds to CD21 on mature B cells to facilitate establishment of latency [71]. It has been shown that gp350 stimulates HERV expression. PBMCs stimulated with EBVgp350 increase expression of MSRV *env* and Syn-1 in a dose-dependent manner, particularly in B cells and monocytes, and even higher in monocyte-derived macrophages [76]. EBVgp350 also induces expression of MSRV *env* and Syn-1 in U87G astrocytes through NF-κB signaling (Figure 3) [76]. There is also indirect evidence for potential links between EBV and ERVs. For example, MSRV *env* is expressed higher in IM patients compared to EBV-negative people and in healthy individuals with high titers of anti-EBNA-1 IgG [77]. More recently, it has been shown that HERV-W *env* expression correlates with EBV load in relapsing–remitting MS patients [78]. In MS patients, the immunosuppressive drugs azathioprine and glatiramer acetate reduce expression of HERV-W env, but whether this involves EBV remains to be tested [76].

HERV-K: The envelope protein of HERV-K18 is encoded in the first intron of CD48 [79] and is a superantigen that activates T cells via TCR Vβ [80]. HERVK-18 was first shown to be induced in primary B cells upon EBV infection [80] and subsequently in EBV-infected human tonsil cells [81]. Mechanistically, this transactivation is mediated by the engagement of EBV gp350 with CD21 on B cells and through the EBV latent membrane proteins (LMPs) LMP-2A and LMP-1, which are expressed downstream of CD21 signaling (Figure 3) [81,82]. LMP-2A-mediated activation of HERV-K18 involves signaling through the immunoreceptor tyrosine-based activation motif (ITAM) of LMP-2A [83]. However, this does not seem to require active viral replication, as overexpression of LMPs and Epstein–Barr nuclear antigens (EBNAs) is sufficient to transactivate HERV-K (HML-2) *gag* [84]. Immortalization by EBV is another cue that makes cells permissive to HERV activation. HERV-K (HML-2) *gag* is upregulated in EBV-immortalized lymphoblastoid cell lines (LCLs) from healthy and MS donors [85]. EBV LMP-2A overexpression transactivates HERV-K18 *env* in EBV-transformed LCLs [82]. Moreover, higher expression of HERV-K *gag*, but not HERV-W *env* or MSRV *env,* has been observed in LCLs derived from MS donors compared to healthy donors, which may or may not involve EBV-associated signaling [85].

### 2.5. Kaposi’s Sarcoma-Associated Herpesvirus (KSHV)

KSHV, also known as HHV-8, is a gammaherpesvirus that infects a broad range of cells and establishes latency in B cells [86]. KSHV is an oncovirus responsible for causing Kaposi’s sarcoma in immunocompromised individuals, most prominently in individuals with acquired immunodeficiency syndrome (AIDS) [87]. Seroprevalence of KSHV varies worldwide, from as high as 80% in sub-Saharan Africa to less than 10% in Asia, northern Europe and the US [88]. It has been reported that HIV+ patients with KSHV express higher levels of HERV-K *env* transcripts than those without KSHV infection [89]. In addition, infection of HUVECs with KSHV or expression of KSHV latency-associated nuclear antigen (LANA) or viral FADD-like interleukin-1-b-converting enzyme inhibitory protein (vFLIP) increases transcription of HERV-K *env*. Activation of HERV-K *env* in the context of KSHV involves the mitogen-activated protein kinase (MAPK) signaling pathway, which is activated by LANA [90], and the transcription factor Sp1, which is activated downstream of receptor tyrosine kinases [89]. It is possible that transactivation of HERVs by KSHV is due to the oncogenic properties of the virus, as other tumor viruses have also been reported to transactivate HERVs [14]. 

### 2.6. Hepatitis B Virus (HBV)

HBV is a hepadnavirus and one of the smallest enveloped viruses with a DNA genome. Following infection of hepatocytes, a covalently closed circular DNA template of the viral genome remains in the nucleus, which can integrate into the host genome [91]. Chronic HBV infection is associated with persistent liver inflammation, fibrosis, hepatocellular carcinoma (HCC) and death. In 2019, the WHO estimated that 296 million people were living with chronic HBV infection, leading to 820,000 deaths. The HBV X protein (HBx) binds to the host genome and plays a role in the pathogenesis of HCC [92,93]. One study showed an NF-κB-dependent increase in HERV-W *env* transcripts and protein in human hepatoma (HepG2) cells expressing HBx [94], but whether HBV infection transactivates HERVs remains to be tested.

## 3. Positive-Sense RNA Viruses

### 3.1. Retroviruses

Human immunodeficiency virus types 1 and 2 (HIV-1, -2) and human T cell lymphotropic virus type 1 (HTLV-1) are positive-sense RNA viruses that require reverse transcription for replication and integration into the host genome [95]. HIV currently affects 39 million individuals worldwide [96], while HTLV-1 infection is rarer, affecting 5 to 10 million individuals. HIV infection causes not just AIDS but also HIV-associated neurocognitive disorders (HANDs) [97], whereas HTLV causes T cell lymphoma. The following studies represent a large body of work showing that HERVs are activated by HIV and HTLV proteins.

#### 3.1.1. HERV Expression in Clinical HIV Samples

An initial study on the plasma of HIV+ individuals showed the presence of HERV-K *pol* in a significantly higher proportion of HIV-1+ individuals compared to uninfected individuals [98]. Analysis of PBMCs from HIV+ individuals, compared to healthy individuals, also showed elevated expression of HERV-K *env* RNA [99] and HERV-K gag protein, particularly in CD4+ and CD8+ T cells [100]. Similarly, HERV-K *gag* is significantly upregulated in the blood of HIV-1 (subtype B)-infected individuals compared to healthy controls [101]. One study showed lower HERV-K expression in those treated with antiretroviral drugs [102], while others have shown elevated HERV-K RNA expression in HIV+ PBMCs, regardless of HIV titers or treatment with highly active antiretroviral drugs (HAART) [102,103]. This raises the possibility that once HERVs are elevated, factor(s) other than HIV promote(s) the continual expression of HERVs. Although most studies do not specify which genomic copies of HERV-K are elevated, HERV-K102 *pol* RNA [104] and several other HERV-K proviral sequences are upregulated in HIV+ individuals compared to healthy people. Finally, HERV-K gag and env proteins along with HER-K viral-like particles are found in the blood of HIV+ patients [105].

#### 3.1.2. HERV Expression in HIV Infection In Vitro

HERV-K: Beyond correlation studies, in vitro HIV infection of both cell lines and primary cells have shown upregulation of HERVs, providing stronger evidence for transactivation of HERVs by HIV. For example, HIV infection of U-87 MG cells results in an increase in HERV-K expression in a dose-dependent manner [100]. This is in contrast to the lack of correlation between viral dose and HERV expression in HIV+ PBMCs. In an H9 T cell line, HERV-K (HML-2) expression is transiently increased within 24 h following HIV infection [100,101]. HIV infection of MT2 and Jurkat T cell lines increases expression of HERV-K *gag* RNA [101,106]. Moreover, HERV-K (HML-3, -4 and -10), along with HERV-E and ERV-9, are upregulated in HIV-infected LC5 cells compared to uninfected LC5 cells [107]. Infection of primary lymphocytes also induces expression of HERV-K *gag* [100] and env RNA and protein [108]. HERV-K loci are also specifically upregulated following HIV infection of CD4+ T cells [109], suggesting selective expression of HERVs in this context.

Additional HERVs: HERV expression has been observed even in astrocytes, which are susceptible to non-productive HIV infection [110,111]. MSRV RNA is increased in brain cell lines, U-87 MG and primary human fetal astrocytes (PHFA) upon HIV infection, while Syn-1 RNA is only upregulated in PHFA [112]. Induction of HERVs in the absence of productive viral replication in these cells may suggest that HIV proteins are sufficient to induce HERV expression, as reviewed in the next section and/or that additional factors are contributing to this phenomenon. Lastly, solo-LTRs like LTR12C are upregulated in primary T cells upon HIV infection [113].

#### 3.1.3. HIV and HTLV Proteins on HERV Expression

Several studies have hinted at the lack of requirement for viral replication in sustaining HERV expression, as is the case for antiretroviral drug-treated individuals and infection of brain cell lines that do not support productive infection. In line with this, there is some evidence that HIV proteins are sufficient to induce HERV expression. For instance, expression of HIV trans-activator of transcription (Tat) alone is sufficient to induce expression of HERV-K *gag* in multiple cell lines including Jurkat T cells, HUT-78 lymphoblasts, U-937 monocytes, 293FT fibroblasts and NCCIT tetracarcinoma cells [106]. This seems to involve a different mechanism than that of the activation of HIV-LTR, as Tat mutants that are incapable of binding to HIV-LTR can still activate the LTR of HERV-K. Tat also acts synergistically with viral infectivity factor (Vif) to induce an even more robust expression of HERV-K *gag* in Jurkat T cells. Stimulation of healthy donor lymphocytes with recombinant Tat results in the expression of 26 HERVs, including HERV-K108 and K115 [114]. Recombinant Tat protein has also been shown to increase transcription of MSRV *env* in primary B cells, natural killer (NK) cells and monocytes, and Syn-1 in B cells. In monocyte-derived macrophages, Tat increases expression of MSRV and Syn-1 through TLR4 signaling [112]. Whether HERV expression always depends on the interaction of Tat with TLR4 [115,116] remains to be determined. Finally, HTLV Tax, a transactivator of viral gene expression [117], can activate promoter activity of HERV-W8, -W18 and HERV-H, HERV-K and HERV-E to varying degrees in Jurkat cells [118]. 

### 3.2. Severe Acute Respiratory Syndrome Coronavirus 2 (SARS-CoV-2)

SARS-CoV-2 is an enveloped positive-sense RNA virus that causes COVID-19 and has infected over 700 million individuals, leading to 7 million deaths worldwide. Approximately 10% of infected individuals experience post-acute sequalae, also known as long COVID, which affects neurological, respiratory, cardiovascular and digestive systems [119].

HERV-W: In COVID-19 patients, HERV-W env RNA and surface protein are elevated in PBMCs, particularly in monocytes, B cells, CD4+ and CD8+ T cells. Heightened expression is observed in both asymptomatic and hospitalized individuals [120]. Another study showed that although HERV-W *env* and HERV-K *env* are elevated in nasal swabs of SARS-CoV-2-positive samples independent of hospitalization status, samples from hospitalized individuals that required oxygen support expressed higher levels [121]. HERV-W env protein has also been detected in the plasma of both acutely infected individuals with moderate to severe disease and those with long COVID [122]. In an in vitro setting, HERV-W env RNA and protein are induced as early as two hours post-infection of PBMCs with SARS-CoV-2. There is also evidence that in vitro stimulation of PBMCs and epithelial cells (FaDu cells) with SARS-CoV-2 spike protein is sufficient to induce HERV-W *env* expression [121,122]. These studies suggest that the SARS-CoV-2 spike protein and early events of infection are enough to cause upregulation of HERV-W, and additional factors associated with severe disease further amplify this effect.

HERV-K: HERV-K *pol* and HERV-H *pol* genes are elevated in the whole blood of children with mildly symptomatic COVID-19 disease, while HERV-W, Syn-1, Syn-2 and MSRV are either unchanged or lower compared to healthy controls [123]. HERV-K *gag* expression is elevated in human monocytes infected with SARS-CoV-2, and this is diminished in the presence of a reverse transcriptase inhibitor (zidovudine, AZT), antiviral (remdesvir, RDV) and anti-inflammatory steroids (dexamethasone and prednisolone) [124]. One study quantified levels of anti-HERV-K env antibody titers in the blood of individuals with post-COVID myalgic encephalomyelitis and chronic fatigue syndrome and showed that higher anti-SARS-CoV-2 Ig titers correlated with higher anti-HERV-K env IgG titers [125]. Although HERV expression was not directly measured, it suggests a link between SARS-CoV-2 levels and HERV reactivity. 

Other HERVs: Locus-specific transcriptome analysis of ERVs showed that HERV-H and HERV-3 elements are elevated in SARS-CoV-2-infected Calu-3 and A549 cells, while HERV-E was commonly elevated in cells infected with SARS-CoV-1, SARS-CoV-2 and Middle East respiratory syndrome coronavirus (MERS) [126]. A number of HERVs are elevated in bronchoalveolar lavage fluids (BALF) from SARS-CoV-2-infected individuals compared to healthy BALF but less so in PBMCs. Similarly, LTR69, an ERV3 element, is elevated in an adenocarcinoma cell line, Calu-3, following SARS-CoV-2-induced H3K27 acetylation. LTR69 expression is also prone to increase following stimulation by polyinosinic:polycytidylic acid (poly:IC) and overexpression of interferon regulatory factor 3 (IRF3) and NF-κB subunit, p65/RELA [127], which are also triggered upon SARS-CoV-2 infection. In contrast, one study showed downregulation of HERV-H, HERV-W and HERV-K in individuals reinfected with SARS-CoV-2 [128].

### 3.3. Dengue Virus (DENV)

DENV is an enveloped positive-strand RNA virus of the *orthoflavivirus* genus consisting of four known serotypes (serotypes 1–4) [129]. Approximately half of the world’s population is at risk of DENV infection, with the highest incidence in Asia. Dengue is a vector-borne disease that usually manifests as a febrile illness, but some patients develop more severe symptoms [130]. Only one study has reported HERV activation by DENV. Using high-throughput RNA sequencing, Wang et al. showed that many HERV loci belonging to the ERV1, ERV2, ERV3 and Gypsy families were differentially expressed in A549 cells infected with DENV serotype 2 [131].

### 3.4. Zika Virus (ZIKV)

ZIKV is also an enveloped positive-strand RNA virus of the *orthoflavivirus* genus. ZIKV is widespread in Africa, Asia and Oceania [132] and caused an epidemic in 2015 and lasting into 2016. While most human infections are asymptomatic, symptomatic patients develop febrile illness and, in some cases, can develop neuroinvasive diseases such as meningitis and encephalitis [133]. Only one study has investigated HERV transactivation by arboviruses. It showed that ZIKV, Mayaro (MayV), Oropouche (OroV) and Chikungunya (ChikV) virus infection of human primary astrocytes upregulates many different families of HERVs. In particular, HERV4_4q22.1 is upregulated to similar levels by all four viruses [134]. 

### 3.5. Hepatitis C Virus (HCV)

HCV causes hepatitis C, a condition where viral persistence in the liver causes inflammation that can lead to liver failure, cirrhosis or HCC. The WHO estimates about 58 million people worldwide have chronic HCV infection, and in 2019, this resulted in 290,000 deaths. Direct-acting antivirals (DAAs) are highly effective against HCV infection, but associated malignancies and autoimmune conditions may develop even after viral clearance [91]. HERV-K, HERV-H and HERV-W *pol* RNA has been found to be elevated in adolescents who were vertically infected with HCV compared to those uninfected [135]. However, reduction in viremia by DAA did not decrease expression of HERV *pol* RNA. HERV-K (HML-2) RNA is elevated in the PBMCs of HCV patients with liver cirrhosis compared to HCV patients without liver cirrhosis, both before and after DAA treatment [136]. In addition, HERV-K is elevated in patients who have an incomplete response to DAA treatment, and this expression correlates with decreased albumin, a marker of impaired liver function. These studies indicate that HERV transactivation may be indirect in this context.

### 3.6. Coxsackievirus (CV)

Coxsackievirus B (CV-B) is a non-enveloped, positive-sense RNA enterovirus that causes gastrointestinal disease. While the virus is usually rapidly cleared, viral mutations can transform the virus into a non-cytolytic or defective form that can persist in tissues [137]. Persistence of serotype CV-B4 in the pancreas has been implicated in diabetes mellitus type 1 (T1D) [138]. HERV-W env RNA and protein levels are upregulated in human pancreatic cells upon infection with CV-B4 compared to uninfected cells [139]. In addition, HERV-W *env* is induced in monocyte-derived macrophages infected with CV-B4 but not in infected PBMCs. It has also been shown that HERV-W *env* is significantly upregulated in the serum of T1D patients compared to controls, and env protein is elevated in the pancreas of T1D patients [140]. Whether HERV-W upregulation in T1D patients is directly dependent on CV-B infection remains to be answered.

## 4. Negative-Sense RNA Viruses

### 4.1. Influenza

Influenza virus is an enveloped virus that belongs to the *Orthomyxoviridae* family and has a segmented, negative-sense RNA genome. Influenza A (IAV) and B (IAB) are two virus types that are responsible for causing epidemics worldwide [141]. Influenza infection in healthy individuals usually resolves on its own, but virus-induced pneumonia can be fatal [142], and highly pathogenic strains of influenza remain a major global concern.

**Figure 4:**
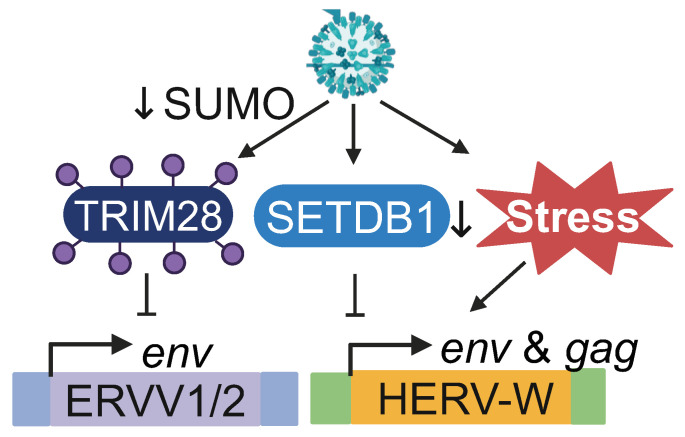
Expression of ERVs during IAV infection. Cellular cues and factors that are modulated during IAV infection regulate expression of ERVV-1, ERVV-2 and HERV-W.

HERV-W: HERV-W *env* and *gag* RNA, including Syn-1, are elevated in human astrocytoma (CCF-STTG1), human histiocytic lymphoma (U937) and kidney epithelioma (293F) cells infected with IAV [48]. In CCF-STTG1 cells, a master transcriptional regulator of syncytiotrophoblast formation called glial cells missing-1 (GSM1) is required for Syn1-encoding *ERVWE1* expression upon IAV infection. Additionally, serum deprivation results in the upregulation of HERV-W *env*, suggesting that cellular stress caused by IAV infection may be involved in the transactivation [48]. Finally, IAV infection causes a reduction in SETDB1 expression as well as H3K9me3 marks on the 5′ LTR and intronic regions of *ERVWE1* (Figure 4), indicating that epigenetic de-silencing of HERV-W during IAV infection is likely mediating the transactivation [143].

Additional HERVs: During IAV infection of lung carcinoma cells (A549) and human fibroblast cells (MRC-5), ERVV-1 and ERVV-2 *env* are elevated and correlate with the loss of small ubiquitin-like modifier (SUMO) linkages on TRIM28 [144]. In fact, SUMOylation-deficient TRIM28 is associated with higher expression of ERVV-1, ERVV-2 and ERV3-1 *env* compared to SUMOylated TRIM28, suggesting that IAV mediates depression of HERVs by targeting SUMO-modified TRIM28 (Figure 4). HERVs belonging to the ERV3 group are also upregulated in A549 cells infected with IAV, and thus differentially expressed HERVs are enriched for the NF-Y transcription factor binding motif within the LTR [145].

### 4.2. Respiratory Syncytial Virus (RSV)

RSV is an enveloped virus with a negative-sense, single-stranded RNA genome. RSV is a major cause of respiratory illness in young children. Most infections cause mild respiratory symptoms, but the involvement of the lower respiratory tract can lead to life-threatening consequences [146]. There are approximately 30 million cases and 100,000 deaths caused by RSV infection worldwide each year [147]. Only one study has investigated the effect of RSV, showing significant downregulation of HERV-H, K, W *pol* and Syn-1 and Syn-2 *env* in the whole blood of children under the age of 3 who were hospitalized with severe RSV bronchiolitis [148]. This is in contrast to the transactivation of HERVs observed in other viral infections, and further investigation is needed to assess whether this is observed in other cohorts.

## 5. Discussion

It is evident that HERVs are activated by a range of DNA and RNA viruses. Although many viruses have not been reported to activate HERV expression, it is likely that some level of HERV transactivation is a universal effect of viral infection. While some studies show clear transactivation of HERVs upon viral infection in vitro, others are more correlative. In some cases, viral load correlates with HERV expression, as in the case of EBV and HCMV, but conflicting evidence exists for others like HIV and SARS-CoV-2. Antiviral treatments may (in the case of HAART of HIV) or may not (DAA treatment of HCV) lead to reduced HERV expression. Some discrepancies also exist when comparing clinical samples to in vitro infection studies, suggesting a need for more mechanistic studies on viral-induced transactivation of HERVs.

Whether HERV activation requires viral infection or not has been probed in multiple studies. Indeed, HERVs are activated by UV-inactivated viruses, stimulation by soluble viral proteins and overexpression of viral proteins, suggesting that viral replication may not be a requirement for transactivation of HERVs. Rather, HERV expression may be a result of early signaling events during infection. Both EBV and HHV-6 infections result in HERV activation through PKC signaling downstream of receptor tyrosine kinases, suggesting that sensing of the extracellular content during infection may potentially play a role. Transcription factors such as NF-κB or Sp1 that are activated during viral infection are also involved in HERV transactivation for HIV, EBV and HBV. Moreover, modulation of epigenetic silencing machinery by viruses like IAV can lead to epigenetic de-silencing of HERVs and subsequent expression, similarly to cancer and autoimmune diseases. Although the exact mechanisms of HERV transactivation in most cases are not known, these studies are beginning to reveal that a combination of intracellular signaling, epigenetic modification and transcriptional activation are involved in the transactivation.

Activation of HERVs is also linked to inflammation in cancer and autoimmunity, reviewed extensively elsewhere [15,149,150,151]. HERVs are also elevated in some cancers that involve viruses, such as human papillomavirus in cervical cancer [152,153,154]. The expression of interferon (IFN-I)-stimulated genes (ISGs) and proinflammatory cytokine genes often correlates with HERV expression in disease. Proximity of differentially expressed genes to HERVs is one contributing factor to this relationship, as observed for DENV and IAV. HERV LTRs in close proximity to antiviral genes can promote transcription of these genes [113]. Moreover, HERV peptides can stimulate T cells to secrete IFN-gamma [99]. The HERV envelope can stimulate innate immune response through TLR4 [67] and can even increase the invasiveness of KSHV-infected HUVEC cells as a result of reduced VEGF signaling [89]. Finally, expression of HERV gag can lead to co-packaging of the HERV gag in newly synthesized HIV virions, resulting in lower replication capacity of HIV—this is also seen in integrase-deficient HIV strains supplemented with HERV-K10 integrase [155]. Together, transactivation of HERV LTR and HERV proteins have downstream consequences that impact the host immune response or replication of exogenous viruses, even through HERVs themselves are not generating replication-complement virions.

There seems to be little specificity in the HERVs that are activated by viral infection, as most viruses activate the expression of HERV-K and HERV-W. However, given the limited number of HERVs that can be assessed by qPCR, compared to the vast number of HERVs that are present in the genome, the absence of signal in existing studies may not reflect biology. On the other hand, some HERVs may never be transcriptionally active due to mutations within the LTR. Further studies using deep sequencing techniques and convergence of methodology to assess the expression of individual LTR and HERV elements will likely provide higher resolution on the specificity of virus–HERV pairs. Finally, more work is needed to further elucidate the dynamics between infectious viruses and HERVs and how this contributes to virus-associated diseases.

## 6. Methods Used to Obtain Papers

We identified articles on viral transactivation of ERVs by searching PubMed. Key words used to search were “human endogenous retrovirus + [name of virus]” to capture the broadest possible range of articles. We included primary research papers showing results from in vitro infections and stimulation with viral proteins and data obtained from clinical samples that were analyzed post-infection. Papers published in languages other than English, conference abstracts or papers without full text availability were not included in the review. We divided and grouped articles by virus to provide a coherent flow of information. Articles on non-human endogenous retroviruses were not included in this review.

## Supplementary Materials

The following supporting information can be downloaded at: https://www.mdpi.com/article/10.3390/v16111649/s1, Table S1: List of differentially expressed ERVs.

## References

[1] Wells J.N., Feschotte C. (2020). A Field Guide to Eukaryotic Transposable Elements. Annu. Rev. Genet..

[2] Jern P., Coffin J.M. (2008). Effects of Retroviruses on Host Genome Function. Annu. Rev. Genet..

[3] Bock M., Stoye J.P. (2000). Endogenous Retroviruses and the Human Germline. Curr. Opin. Genet. Dev..

[4] Gifford R.J. (2012). Viral Evolution in Deep Time: Lentiviruses and Mammals. Trends Genet..

[5] Lander E.S., Linton L.M., Birren B., Nusbaum C., Zody M.C., Baldwin J., Devon K., Dewar K., Doyle M., FitzHugh W. (2001). Initial Sequencing and Analysis of the Human Genome. Nature.

[6] Johnson W.E. (2019). Origins and Evolutionary Consequences of Ancient Endogenous Retroviruses. Nat. Rev. Microbiol..

[7] Stoye J.P. (2012). Studies of Endogenous Retroviruses Reveal a Continuing Evolutionary Saga. Nat. Rev. Microbiol..

[8] Kassiotis G. (2023). The Immunological Conundrum of Endogenous Retroelements. Annu. Rev. Immunol..

[9] Grandi N., Tramontano E. (2018). HERV Envelope Proteins: Physiological Role and Pathogenic Potential in Cancer and Autoimmunity. Front. Microbiol..

[10] Küry P., Nath A., Créange A., Dolei A., Marche P., Gold J., Giovannoni G., Hartung H.-P., Perron H. (2018). Human Endogenous Retroviruses in Neurological Diseases. Trends Mol. Med..

[11] Jansz N., Faulkner G.J. (2021). Endogenous Retroviruses in the Origins and Treatment of Cancer. Genome Biol..

[12] Bannert N., Hofmann H., Block A., Hohn O. (2018). HERVs New Role in Cancer: From Accused Perpetrators to Cheerful Protectors. Front. Microbiol..

[13] Badarinarayan S.S., Sauter D. (2021). Switching Sides: How Endogenous Retroviruses Protect Us from Viral Infections. J. Virol..

[14] Chen J., Foroozesh M., Qin Z. (2019). Transactivation of Human Endogenous Retroviruses by Tumor Viruses and Their Functions in Virus-Associated Malignancies. Oncogenesis.

[15] Dopkins N., Nixon D.F. (2024). Activation of Human Endogenous Retroviruses and Its Physiological Consequences. Nat. Rev. Mol. Cell Biol..

[16] Baker R.E., Mahmud A.S., Miller I.F., Rajeev M., Rasambainarivo F., Rice B.L., Takahashi S., Tatem A.J., Wagner C.E., Wang L.-F. (2022). Infectious Disease in an Era of Global Change. Nat. Rev. Microbiol..

[17] Habibi M.A., Shamohammadi F.N., Rajaei T., Namdari H., Pashaei M.R., Farajifard H., Ahmadpour S. (2023). Immunopathogenesis of Viral Infections in Neurological Autoimmune Disease. BMC Neurol..

[18] Sundaresan B., Shirafkan F., Ripperger K., Rattay K. (2023). The Role of Viral Infections in the Onset of Autoimmune Diseases. Viruses.

[19] Fujinami R.S. (2001). Viruses and Autoimmune Disease—Two Sides of the Same Coin?. Trends Microbiol..

[20] Bannert N., Kurth R. (2006). The Evolutionary Dynamics of Human Endogenous Retroviral Families. Annu. Rev. Genom. Hum. Genet..

[21] Gemmell P., Hein J., Katzourakis A. (2019). The Exaptation of HERV-H: Evolutionary Analyses Reveal the Genomic Features of Highly Transcribed Elements. Front. Immunol..

[22] Gemmell P., Hein J., Katzourakis A. (2016). Phylogenetic Analysis Reveals That ERVs “Die Young” but HERV-H Is Unusually Conserved. PLOS Comput. Biol..

[23] Mager D.L., Henthorn P.S. (1984). Identification of a Retrovirus-like Repetitive Element in Human DNA. Proc. Natl. Acad. Sci. USA.

[24] Blond J.-L., Besème F., Duret L., Bouton O., Bedin F., Perron H., Mandrand B., Mallet F. (1999). Molecular Characterization and Placental Expression of HERV-W, a New Human Endogenous Retrovirus Family. J. Virol..

[25] Blond J.-L., Lavillette D., Cheynet V., Bouton O., Oriol G., Chapel-Fernandes S., Mandrand B., Mallet F., Cosset F.-L. (2000). An Envelope Glycoprotein of the Human Endogenous Retrovirus HERV-W Is Expressed in the Human Placenta and Fuses Cells Expressing the Type D Mammalian Retrovirus Receptor. J. Virol..

[26] Mi S., Lee X., Li X., Veldman G.M., Finnerty H., Racie L., LaVallie E., Tang X.-Y., Edouard P., Howes S. (2000). Syncytin Is a Captive Retroviral Envelope Protein Involved in Human Placental Morphogenesis. Nature.

[27] Jha A.R., Nixon D.F., Rosenberg M.G., Martin J.N., Deeks S.G., Hudson R.R., Garrison K.E., Pillai S.K. (2011). Human Endogenous Retrovirus K106 (HERV-K106) Was Infectious after the Emergence of Anatomically Modern Humans. PLoS ONE.

[28] Marchi E., Kanapin A., Byott M., Magiorkinis G., Belshaw R. (2013). Neanderthal and Denisovan Retroviruses in Modern Humans. Curr. Biol..

[29] Marta G.-M., Tara D.-O., Lisa H., Avindra N. (2019). Human Endogenous Retrovirus-K (HML-2): A Comprehensive Review. Crit. Rev. Microbiol..

[30] MOYES D., MARTIN A., SAWCER S., TEMPERTON N., WORTHINGTON J., GRIFFITHS D., VENABLES P. (2005). The Distribution of the Endogenous Retroviruses HERV-K113 and HERV-K115 in Health and Disease. Genomics.

[31] Turner G., Barbulescu M., Su M., Jensen-Seaman M.I., Kidd K.K., Lenz J. (2001). Insertional Polymorphisms of Full-Length Endogenous Retroviruses in Humans. Curr. Biol..

[32] Wildschutte J.H., Williams Z.H., Montesion M., Subramanian R.P., Kidd J.M., Coffin J.M. (2016). Discovery of Unfixed Endogenous Retrovirus Insertions in Diverse Human Populations. Proc. Natl. Acad. Sci. USA.

[33] Flockerzi A., Ruggieri A., Frank O., Sauter M., Maldener E., Kopper B., Wullich B., Seifarth W., Müller-Lantzsch N., Leib-Mösch C. (2008). Expression Patterns of Transcribed Human Endogenous Retrovirus HERV-K(HML-2) Loci in Human Tissues and the Need for a HERV Transcriptome Project. BMC Genom..

[34] Burn A., Roy F., Freeman M., Coffin J.M. (2022). Widespread Expression of the Ancient HERV-K (HML-2) Provirus Group in Normal Human Tissues. PLoS Biol..

[35] Pérot P., Mugnier N., Montgiraud C., Gimenez J., Jaillard M., Bonnaud B., Mallet F. (2012). Microarray-Based Sketches of the HERV Transcriptome Landscape. PLoS ONE.

[36] Ito J., Sugimoto R., Nakaoka H., Yamada S., Kimura T., Hayano T., Inoue I. (2017). Systematic Identification and Characterization of Regulatory Elements Derived from Human Endogenous Retroviruses. PLoS Genet..

[37] Rowe H.M., Trono D. (2011). Dynamic Control of Endogenous Retroviruses during Development. Virology.

[38] Imbeault M., Helleboid P.-Y., Trono D. (2017). KRAB Zinc-Finger Proteins Contribute to the Evolution of Gene Regulatory Networks. Nature.

[39] Wolf G., Greenberg D., Macfarlan T.S. (2015). Spotting the Enemy within: Targeted Silencing of Foreign DNA in Mammalian Genomes by the Krüppel-Associated Box Zinc Finger Protein Family. Mob. DNA.

[40] Geis F.K., Goff S.P. (2020). Silencing and Transcriptional Regulation of Endogenous Retroviruses: An Overview. Viruses.

[41] Perron H., Suh M., Lalande B., Gratacap B., Laurent A., Stoebner P., Seigneurin J.M. (1993). Herpes Simplex Virus ICP0 and ICP4 Immediate Early Proteins Strongly Enhance Expression of a Retrovirus Harboured by a Leptomeningeal Cell Line from a Patient with Multiple Sclerosis. J. Gen. Virol..

[42] World Health Organization Herpes Simplex Virus. https://www.who.int/news-room/fact-sheets/detail/herpes-simplex-virus.

[43] Whitley R.J., Baron S. (1996). Chapter 68 Herpesviruses. Medical Microbiology.

[44] Ferrante P., Mancuso R., Pagani E., Guerini F.R., Calvo M.G., Saresella M., Speciale L., Caputo D. (2000). Molecular Evidences for a Role of HSV-1 in Multiple Sclerosis Clinical Acute Attack. J. Neurovirology.

[45] Duarte L.F., Altamirano-Lagos M.J., Tabares-Guevara J.H., Opazo M.C., Díaz M., Navarrete R., Muza C., Vallejos O.P., Riedel C.A., Bueno S.M. (2021). Asymptomatic Herpes Simplex Virus Type 1 Infection Causes an Earlier Onset and More Severe Experimental Autoimmune Encephalomyelitis. Front. Immunol..

[46] Perron H., Geny C., Laurent A., Mouriquand C., Pellat J., Perret J., Seigneurin J.M. (1989). Leptomeningeal Cell Line from Multiple Sclerosis with Reverse Transcriptase Activity and Viral Particles. Res. Virol..

[47] Lee W.J., Kwun H.J., Kim H.S., Jang K.L. (2003). Activation of the Human Endogenous Retrovirus W Long Terminal Repeat by Herpes Simplex Virus Type 1 Immediate Early Protein 1. Mol. Cells.

[48] Nellåker C., Yao Y., Jones-Brando L., Mallet F., Yolken R.H., Karlsson H. (2006). Transactivation of Elements in the Human Endogenous Retrovirus W Family by Viral Infection. Retrovirology.

[49] Ruprecht K., Obojes K., Wengel V., Gronen F., Kim K.S., Perron H., Schneider-Schaulies J., Rieckmann P. (2006). Regulation of Human Endogenous Retrovirus W Protein Expression by Herpes Simplex Virus Type 1: Implications for Multiple Sclerosis. J. NeuroVirology.

[50] Brudek T., Lühdorf P., Christensen T., Hansen H.J., Møller-Larsen A. (2007). Activation of Endogenous Retrovirus Reverse Transcriptase in Multiple Sclerosis Patient Lymphocytes by Inactivated HSV-1, HHV-6 and VZV. J. Neuroimmunol..

[51] Kwun H.J., Han H.J., Lee W.J., Kim H.S., Jang K.L. (2002). Transactivation of the Human Endogenous Retrovirus K Long Terminal Repeat by Herpes Simplex Virus Type 1 Immediate Early Protein 0. Virus Res..

[52] Zuhair M., Smit G.S.A., Wallis G., Jabbar F., Smith C., Devleesschauwer B., Griffiths P. (2019). Estimation of the Worldwide Seroprevalence of Cytomegalovirus: A Systematic Review and Meta-analysis. Rev. Med. Virol..

[53] Forte E., Zhang Z., Thorp E.B., Hummel M. (2020). Cytomegalovirus Latency and Reactivation: An Intricate Interplay With the Host Immune Response. Front. Cell. Infect. Microbiol..

[54] Griffiths P., Reeves M. (2021). Pathogenesis of Human Cytomegalovirus in the Immunocompromised Host. Nat. Rev. Microbiol..

[55] Nelson P.N., Lever A.M.L., Smith S., Pitman R., Murray P., Perera S.A., Westwood O.M.R., Hay F.C., Ejtehadi H.D., Booth J.C. (1999). Molecular Investigations Implicate Human Endogenous Retroviruses as Mediators of Anti-Retroviral Antibodies in Autoimmune Rheumatic Disease. Immunol. Investig..

[56] Bergallo M., Galliano I., Montanari P., Gambarino S., Mareschi K., Ferro F., Fagioli F., Tovo P.-A., Ravanini P. (2015). CMV Induces HERV-K and HERV-W Expression in Kidney Transplant Recipients. J. Clin. Virol..

[57] Assinger A., Yaiw K.-C., Göttesdorfer I., Leib-Mösch C., Söderberg-Nauclér C. (2013). Human Cytomegalovirus (HCMV) Induces Human Endogenous Retrovirus (HERV) Transcription. Retrovirology.

[58] Esteki-Zadeh A., Karimi M., Strååt K., Ammerpohl O., Zeitelhofer M., Jagodic M., Mehrab-Mohseni M., Sjöholm L., Rahbar A., Söderberg-Nauclér C. (2012). Human Cytomegalovirus Infection Is Sensitive to the Host Cell DNA Methylation State and Alters Global DNA Methylation Capacity. Epigenetics.

[59] Luppi M., Barozzi P., Morris C., Maiorana A., Garber R., Bonacorsi G., Donelli A., Marasca R., Tabilio A., Torelli G. (1999). Human Herpesvirus 6 Latently Infects Early Bone Marrow Progenitors In Vivo. J. Virol..

[60] Luppi M., Barozzi P., Maiorana A., Marasca R., Torelli G. (1994). Human Herpesvirus 6 Infection in Normal Human Brain Tissue. J. Infect. Dis..

[61] Tesini B.L., Epstein L.G., Caserta M.T. (2014). Clinical Impact of Primary Infection with Roseoloviruses. Curr. Opin. Virol..

[62] Hall C.B., Long C.E., Schnabel K.C., Caserta M.T., McIntyre K.M., Costanzo M.A., Knott A., Dewhurst S., Insel R.A., Epstein L.G. (1994). Human Herpesvirus-6 Infection in Children—A Prospective Study of Complications and Reactivation. N. Engl. J. Med..

[63] Leibovitch E.C., Jacobson S. (2014). Evidence Linking HHV-6 with Multiple Sclerosis: An Update. Curr. Opin. Virol..

[64] Caselli E., Zatelli M.C., Rizzo R., Benedetti S., Martorelli D., Trasforini G., Cassai E., Uberti E.C.D., Luca D.D., Dolcetti R. (2012). Virologic and Immunologic Evidence Supporting an Association between HHV-6 and Hashimoto’s Thyroiditis. PLoS Pathog..

[65] Ablashi D.V., Devin C.L., Yoshikawa T., Lautenschlager I., Luppi M., Kühl U., Komaroff A.L. (2010). Review Part 3: Human Herpesvirus-6 in Multiple Non-neurological Diseases. J. Med. Virol..

[66] Santoro F., Kennedy P.E., Locatelli G., Malnati M.S., Berger E.A., Lusso P. (1999). CD46 Is a Cellular Receptor for Human Herpesvirus 6. Cell.

[67] Charvet B., Reynaud J.M., Gourru-Lesimple G., Perron H., Marche P.N., Horvat B. (2018). Induction of Proinflammatory Multiple Sclerosis-Associated Retrovirus Envelope Protein by Human Herpesvirus-6A and CD46 Receptor Engagement. Front. Immunol..

[68] Tai A.K., Luka J., Ablashi D., Huber B.T. (2009). HHV-6A Infection Induces Expression of HERV-K18-Encoded Superantigen. J. Clin. Virol..

[69] Turcanova V.L., Bundgaard B., Höllsberg P. (2009). Human Herpesvirus-6B Induces Expression of the Human Endogenous Retrovirus K18-Encoded Superantigen. J. Clin. Virol..

[70] Shannon-Lowe C., Rowe M. (2014). Epstein Barr Virus Entry; Kissing and Conjugation. Curr. Opin. Virol..

[71] Damania B., Kenney S.C., Raab-Traub N. (2022). Epstein-Barr Virus: Biology and Clinical Disease. Cell.

[72] Khan G., Fitzmaurice C., Naghavi M., Ahmed L.A. (2020). Global and Regional Incidence, Mortality and Disability-Adjusted Life-Years for Epstein-Barr Virus-Attributable Malignancies, 1990–2017. BMJ Open.

[73] Moreno M.A., Or-Geva N., Aftab B.T., Khanna R., Croze E., Steinman L., Han M.H. (2018). Molecular Signature of Epstein-Barr Virus Infection in MS Brain Lesions. Neurol. Neuroimmunol. Neuroinflammation.

[74] Bjornevik K., Cortese M., Healy B.C., Kuhle J., Mina M.J., Leng Y., Elledge S.J., Niebuhr D.W., Scher A.I., Munger K.L. (2022). Longitudinal Analysis Reveals High Prevalence of Epstein-Barr Virus Associated with Multiple Sclerosis. Science.

[75] Goldacre R. (2024). Risk of Multiple Sclerosis in Individuals with Infectious Mononucleosis: A National Population-Based Cohort Study Using Hospital Records in England, 2003–2023. Mult. Scler. J..

[76] Mameli G., Poddighe L., Mei A., Uleri E., Sotgiu S., Serra C., Manetti R., Dolei A. (2012). Expression and Activation by Epstein Barr Virus of Human Endogenous Retroviruses-W in Blood Cells and Astrocytes: Inference for Multiple Sclerosis. PLoS ONE.

[77] Mameli G., Madeddu G., Mei A., Uleri E., Poddighe L., Delogu L.G., Maida I., Babudieri S., Serra C., Manetti R. (2013). Activation of MSRV-Type Endogenous Retroviruses during Infectious Mononucleosis and Epstein-Barr Virus Latency: The Missing Link with Multiple Sclerosis?. PLoS ONE.

[78] Pérez-Pérez S., Domínguez-Mozo M.I., García-Martínez M.Á., Ballester-González R., Nieto-Gañán I., Arroyo R., Alvarez-Lafuente R. (2022). Epstein-Barr Virus Load Correlates with Multiple Sclerosis-Associated Retrovirus Envelope Expression. Biomedicines.

[79] Hasuike S., Miura K., Miyoshi O., Miyamoto T., Niikawa N., Jinno Y., Ishikawa M. (1999). Isolation and Localization of an IDDMK1,2-22-Related Human Endogenous Retroviral Gene, and Identification of a CA Repeat Marker at Its Locus. J. Hum. Genet..

[80] Sutkowski N., Conrad B., Thorley-Lawson D.A., Huber B.T. (2001). Epstein-Barr Virus Transactivates the Human Endogenous Retrovirus HERV-K18 That Encodes a Superantigen. Immunity.

[81] Hsiao F.C., Lin M., Tai A., Chen G., Huber B.T. (2006). Cutting Edge: Epstein-Barr Virus Transactivates the HERV-K18 Superantigen by Docking to the Human Complement Receptor 2 (CD21) on Primary B Cells. J. Immunol..

[82] Sutkowski N., Chen G., Calderon G., Huber B.T. (2004). Epstein-Barr Virus Latent Membrane Protein LMP-2A Is Sufficient for Transactivation of the Human Endogenous Retrovirus HERV-K18 Superantigen. J. Virol..

[83] Hsiao F.C., Tai A.K., Deglon A., Sutkowski N., Longnecker R., Huber B.T. (2009). EBV LMP-2A Employs a Novel Mechanism to Transactivate the HERV-K18 Superantigen through Its ITAM. Virology.

[84] Freimanis G., Hooley P., Ejtehadi H.D., Ali H.A., Veitch A., Rylance P.B., Alawi A., Axford J., Nevill A., Murray P.G. (2010). A Role for Human Endogenous Retrovirus-K (HML-2) in Rheumatoid Arthritis: Investigating Mechanisms of Pathogenesis. Clin. Exp. Immunol..

[85] Wieland L., Schwarz T., Engel K., Volkmer I., Krüger A., Tarabuko A., Junghans J., Kornhuber M.E., Hoffmann F., Staege M.S. (2022). Epstein-Barr Virus-Induced Genes and Endogenous Retroviruses in Immortalized B Cells from Patients with Multiple Sclerosis. Cells.

[86] Szymula A., Samayoa-Reyes G., Ogolla S., Liu B., Li S., George A., Sciver N.V., Rochford R., Simas J.P., Kaye K.M. (2023). Macrophages Drive KSHV B Cell Latency. Cell Rep..

[87] Cesarman E., Damania B., Krown S.E., Martin J., Bower M., Whitby D. (2019). Kaposi Sarcoma. Nat. Rev. Dis. Prim..

[88] Chatlynne L.G., Ablashi D.V. (1999). Seroepidemiology of Kaposi’s Sarcoma-Associated Herpesvirus (KSHV). Semin. Cancer Biol..

[89] Dai L., Valle L.D., Miley W., Whitby D., Ochoa A.C., Flemington E.K., Qin Z. (2018). Transactivation of Human Endogenous Retrovirus K (HERV-K) by KSHV Promotes Kaposi’s Sarcoma Development. Oncogene.

[90] Roupelieva M., Griffiths S.J., Kremmer E., Meisterernst M., Viejo-Borbolla A., Schulz T., Haas J. (2010). Kaposi’s Sarcoma-Associated Herpesvirus Lana-1 Is a Major Activator of the Serum Response Element and Mitogen-Activated Protein Kinase Pathways via Interactions with the Mediator Complex. J. Gen. Virol..

[91] Saraceni C., Birk J. (2021). A Review of Hepatitis B Virus and Hepatitis C Virus Immunopathogenesis. J. Clin. Transl. Hepatol..

[92] Levrero M., Zucman-Rossi J. (2016). Mechanisms of HBV-Induced Hepatocellular Carcinoma. J. Hepatol..

[93] Guerrieri F., Belloni L., D’Andrea D., Pediconi N., Pera L.L., Testoni B., Scisciani C., Floriot O., Zoulim F., Tramontano A. (2017). Genome-Wide Identification of Direct HBx Genomic Targets. BMC Genom..

[94] Liu C., Liu L., Wang X., Liu Y., Wang M., Zhu F. (2017). HBV X Protein Induces Overexpression of HERV-W Env through NF-ΚB in HepG2 Cells. Virus Genes.

[95] Vogt V., Coffin J.M., Hughes S.H., Varmus H.E. (1997). Retroviruses: Retroviral Virions and Genomes.

[96] World Health Organization HIV and AIDS 2024. https://www.who.int/news-room/fact-sheets/detail/hiv-aids.

[97] McArthur J.C., Steiner J., Sacktor N., Nath A. (2010). Human Immunodeficiency Virus-associated Neurocognitive Disorders: Mind the Gap. Ann. Neurol..

[98] Contreras-Galindo R., Kaplan M.H., Markovitz D.M., Lorenzo E., Yamamura Y. (2006). Detection of HERV-K(HML-2) Viral RNA in Plasma of HIV Type 1-Infected Individuals. AIDS Res. Hum. Retroviruses.

[99] Garrison K.E., Jones R.B., Meiklejohn D.A., Anwar N., Ndhlovu L.C., Chapman J.M., Erickson A.L., Agrawal A., Spotts G., Hecht F.M. (2007). T Cell Responses to Human Endogenous Retroviruses in HIV-1 Infection. PLoS Pathog..

[100] Contreras-Galindo R., Lpez P., Vlez R., Yamamura Y. (2007). HIV-1 Infection Increases the Expression of Human Endogenous Retroviruses Type K (HERV-K) In Vitro. AIDS Res. Hum. Retroviruses.

[101] Li X., Guo Y., Li H., Huang X., Pei Z., Wang X., Liu Y., Jia L., Li T., Bao Z. (2021). Infection by Diverse HIV-1 Subtypes Leads to Different Elevations in HERV-K Transcriptional Levels in Human T Cell Lines. Front. Microbiol..

[102] Contreras-Galindo R., Almodvar-Camacho S., Gonzlez-Ramrez S., Lorenzo E., Yamamura Y. (2007). Short Communication Comparative Longitudinal Studies of HERV-K and HIV-1 RNA Titers in HIV-1-Infected Patients Receiving Successful versus Unsuccessful Highly Active Antiretroviral Therapy. AIDS Res. Hum. Retroviruses.

[103] Bhardwaj N., Maldarelli F., Mellors J., Coffin J.M. (2014). HIV-1 Infection Leads to Increased Transcription of Human Endogenous Retrovirus HERV-K (HML-2) Proviruses In Vivo but Not to Increased Virion Production. J. Virol..

[104] Laderoute M.P., Giulivi A., Larocque L., Bellfoy D., Hou Y., Wu H.-X., Fowke K., Wu J., Diaz-Mitoma F. (2007). The Replicative Activity of Human Endogenous Retrovirus K102 (HERV-K102) with HIV Viremia. AIDS.

[105] Contreras-Galindo R., Kaplan M.H., Contreras-Galindo A.C., Gonzalez-Hernandez M.J., Ferlenghi I., Giusti F., Lorenzo E., Gitlin S.D., Dosik M.H., Yamamura Y. (2012). Characterization of Human Endogenous Retroviral Elements in the Blood of HIV-1-Infected Individuals. J. Virol..

[106] Gonzalez-Hernandez M.J., Swanson M.D., Contreras-Galindo R., Cookinham S., King S.R., Noel R.J., Kaplan M.H., Markovitz D.M. (2012). Expression of Human Endogenous Retrovirus Type K (HML-2) Is Activated by the Tat Protein of HIV-1. J. Virol..

[107] Vincendeau M., Göttesdorfer I., Schreml J.M.H., Wetie A.G.N., Mayer J., Greenwood A.D., Helfer M., Kramer S., Seifarth W., Hadian K. (2015). Modulation of Human Endogenous Retrovirus (HERV) Transcription during Persistent and de Novo HIV-1 Infection. Retrovirology.

[108] Michaud H.-A., Mulder M.D., SenGupta D., Deeks S.G., Martin J.N., Pilcher C.D., Hecht F.M., Sacha J.B., Nixon D.F. (2014). Trans-Activation, Post-Transcriptional Maturation, and Induction of Antibodies to HERV-K (HML-2) Envelope Transmembrane Protein in HIV-1 Infection. Retrovirology.

[109] Young G.R., Terry S.N., Manganaro L., Cuesta-Dominguez A., Deikus G., Bernal-Rubio D., Campisi L., Fernandez-Sesma A., Sebra R., Simon V. (2018). HIV-1 Infection of Primary CD4+ T Cells Regulates the Expression of Specific Human Endogenous Retrovirus HERV-K (HML-2) Elements. J. Virol..

[110] Gorry P.R., Howard J.L., Churchill M.J., Anderson J.L., Cunningham A., Adrian D., McPhee D.A., Purcell D.F.J. (1999). Diminished Production of Human Immunodeficiency Virus Type 1 in Astrocytes Results from Inefficient Translation of Gag, Env, and Nef MRNAs despite Efficient Expression of Tat and Rev. J. Virol..

[111] Valdebenito S., Castellano P., Ajasin D., Eugenin E.A. (2021). Astrocytes Are HIV Reservoirs in the Brain: A Cell Type with Poor HIV Infectivity and Replication but Efficient Cell-to-cell Viral Transfer. J. Neurochem..

[112] Uleri E., Mei A., Mameli G., Poddighe L., Serra C., Dolei A. (2014). HIV Tat Acts on Endogenous Retroviruses of the W Family and This Occurs via Toll-like Receptor 4. AIDS.

[113] Badarinarayan S.S., Shcherbakova I., Langer S., Koepke L., Preising A., Hotter D., Kirchhoff F., Sparrer K.M.J., Schotta G., Sauter D. (2020). HIV-1 Infection Activates Endogenous Retroviral Promoters Regulating Antiviral Gene Expression. Nucleic Acids Res..

[114] Gonzalez-Hernandez M.J., Cavalcoli J.D., Sartor M.A., Contreras-Galindo R., Meng F., Dai M., Dube D., Saha A.K., Gitlin S.D., Omenn G.S. (2014). Regulation of the Human Endogenous Retrovirus K (HML-2) Transcriptome by the HIV-1 Tat Protein. J. Virol..

[115] Ali A., Mishra R., Kaur H., Banerjea A.C. (2021). HIV-1 Tat: An Update on Transcriptional and Non-Transcriptional Functions. Biochimie.

[116] Haij N.B., Leghmari K., Planès R., Thieblemont N., Bahraoui E. (2013). HIV-1 Tat Protein Binds to TLR4-MD2 and Signals to Induce TNF-α and IL-10. Retrovirology.

[117] Mohanty S., Harhaj E.W. (2020). Mechanisms of Oncogenesis by HTLV-1 Tax. Pathogens.

[118] Toufaily C., Landry S., Leib-Mosch C., Rassart E., Barbeau B. (2011). Activation of LTRs from Different Human Endogenous Retrovirus (HERV) Families by the HTLV-1 Tax Protein and T-Cell Activators. Viruses.

[119] Davis H.E., McCorkell L., Vogel J.M., Topol E.J. (2023). Long COVID: Major Findings, Mechanisms and Recommendations. Nat. Rev. Microbiol..

[120] Balestrieri E., Minutolo A., Petrone V., Fanelli M., Iannetta M., Malagnino V., Zordan M., Vitale P., Charvet B., Horvat B. (2021). Evidence of the Pathogenic HERV-W Envelope Expression in T Lymphocytes in Association with the Respiratory Outcome of COVID-19 Patients. eBioMedicine.

[121] Petrone V., Fanelli M., Giudice M., Toschi N., Conti A., Maracchioni C., Iannetta M., Resta C., Cipriani C., Miele M.T. (2023). Expression Profile of HERVs and Inflammatory Mediators Detected in Nasal Mucosa as a Predictive Biomarker of COVID-19 Severity. Front. Microbiol..

[122] Charvet B., Brunel J., Pierquin J., Iampietro M., Decimo D., Queruel N., Lucas A., del Mar Encabo-Berzosa M., Arenaz I., Marmolejo T.P. (2023). SARS-CoV-2 Awakens Ancient Retroviral Genes and the Expression of Proinflammatory HERV-W Envelope Protein in COVID-19 Patients. iScience.

[123] Tovo P.-A., Garazzino S., Daprà V., Pruccoli G., Calvi C., Mignone F., Alliaudi C., Denina M., Scolfaro C., Zoppo M. (2021). COVID-19 in Children: Expressions of Type I/II/III Interferons, TRIM28, SETDB1, and Endogenous Retroviruses in Mild and Severe Cases. Int. J. Mol. Sci..

[124] Temerozo J.R., Fintelman-Rodrigues N., dos Santos M.C., Hottz E.D., Sacramento C.Q., Silva A.d.P.D.d., Mandacaru S.C., Moraes E.C.d.S., Trugilho M.R.O., Gesto J.S.M. (2022). Human Endogenous Retrovirus K in the Respiratory Tract Is Associated with COVID-19 Physiopathology. Microbiome.

[125] Apostolou E., Rizwan M., Moustardas P., Sjögren P., Bertilson B.C., Bragée B., Polo O., Rosén A. (2022). Saliva Antibody-Fingerprint of Reactivated Latent Viruses after Mild/Asymptomatic COVID-19 Is Unique in Patients with Myalgic-Encephalomyelitis/Chronic Fatigue Syndrome. Front. Immunol..

[126] Marston J.L., Greenig M., Singh M., Bendall M.L., Duarte R.R.R., Feschotte C., Iñiguez L.P., Nixon D.F. (2021). SARS-CoV-2 Infection Mediates Differential Expression of Human Endogenous Retroviruses and Long Interspersed Nuclear Elements. JCI Insight.

[127] Arora A., Kolberg J.E., Badarinarayan S.S., Savytska N., Munot D., Müller M., Krchlíková V., Sauter D., Bansal V. (2023). SARS-CoV-2 Infection Induces Epigenetic Changes in the LTR69 Subfamily of Endogenous Retroviruses. Mob. DNA.

[128] Grandi N., Erbì M.C., Scognamiglio S., Tramontano E. (2023). Human Endogenous Retrovirus (HERV) Transcriptome Is Dynamically Modulated during SARS-CoV-2 Infection and Allows Discrimination of COVID-19 Clinical Stages. Microbiol. Spectr..

[129] Guzman M.G., Gubler D.J., Izquierdo A., Martinez E., Halstead S.B. (2016). Dengue Infection. Nat. Rev. Dis. Primers.

[130] Bhatt S., Gething P.W., Brady O.J., Messina J.P., Farlow A.W., Moyes C.L., Drake J.M., Brownstein J.S., Hoen A.G., Sankoh O. (2013). The Global Distribution and Burden of Dengue. Nature.

[131] Wang M., Qiu Y., Liu H., Liang B., Fan B., Zhou X., Liu D. (2020). Transcription Profile of Human Endogenous Retroviruses in Response to Dengue Virus Serotype 2 Infection. Virology.

[132] Plourde A.R., Bloch E.M. (2016). A Literature Review of Zika Virus. Emerg. Infect. Dis..

[133] Salimi H., Cain M.D., Klein R.S. (2016). Encephalitic Arboviruses: Emergence, Clinical Presentation, and Neuropathogenesis. Neurotherapeutics.

[134] De Castro F.L., Brustolini O.J.B., Geddes V.E.V., de Souza J.P.B.M., Alves-Leon S.V., Aguiar R.S., Vasconcelos A.T.R. (2022). Modulation of HERV Expression by Four Different Encephalitic Arboviruses during Infection of Human Primary Astrocytes. Viruses.

[135] Tovo P.-A., Garazzino S., Daprà V., Alliaudi C., Silvestro E., Calvi C., Montanari P., Galliano I., Bergallo M. (2020). Chronic HCV Infection Is Associated with Overexpression of Human Endogenous Retroviruses That Persists after Drug-Induced Viral Clearance. Int. J. Mol. Sci..

[136] Weber M., Nair V.P., Bauer T., Sprinzl M.F., Protzer U., Vincendeau M. (2021). Increased HERV-K(HML-2) Transcript Levels Correlate with Clinical Parameters of Liver Damage in Hepatitis C Patients. Cells.

[137] Chapman, Nora M. (2022). Persistent Enterovirus Infection: Little Deletions, Long Infections. Vaccines.

[138] Carré A., Vecchio F., Flodström-Tullberg M., You S., Mallone R. (2023). Coxsackievirus and Type 1 Diabetes: Diabetogenic Mechanisms and Implications for Prevention. Endocr. Rev..

[139] Dechaumes A., Bertin A., Sane F., Levet S., Varghese J., Charvet B., Gmyr V., Kerr-Conte J., Pierquin J., Arunkumar G. (2020). Coxsackievirus-B4 Infection Can Induce the Expression of Human Endogenous Retrovirus W in Primary Cells. Microorganisms.

[140] Levet S., Medina J., Joanou J., Demolder A., Queruel N., Réant K., Normand M., Seffals M., Dimier J., Germi R. (2017). An Ancestral Retroviral Protein Identified as a Therapeutic Target in Type-1 Diabetes. JCI Insight.

[141] Uyeki T.M., Hui D.S., Zambon M., Wentworth D.E., Monto A.S. (2022). Influenza. Lancet.

[142] Taubenberger J.K., Morens D.M. (2008). The Pathology of Influenza Virus Infections. Annu. Rev. Pathol. Mech. Dis..

[143] Li F., Nellåker C., Sabunciyan S., Yolken R.H., Jones-Brando L., Johansson A.-S., Owe-Larsson B., Karlsson H. (2014). Transcriptional Derepression of the ERVWE1 Locus Following Influenza A Virus Infection. J. Virol..

[144] Schmidt N., Domingues P., Golebiowski F., Patzina C., Tatham M.H., Hay R.T., Hale B.G. (2019). An Influenza Virus-Triggered SUMO Switch Orchestrates Co-Opted Endogenous Retroviruses to Stimulate Host Antiviral Immunity. Proc. Natl. Acad. Sci. USA.

[145] Liu H., Bergant V., Frishman G., Ruepp A., Pichlmair A., Vincendeau M., Frishman D. (2022). Influenza A Virus Infection Reactivates Human Endogenous Retroviruses Associated with Modulation of Antiviral Immunity. Viruses.

[146] Borchers A.T., Chang C., Gershwin M.E., Gershwin L.J. (2013). Respiratory Syncytial Virus—A Comprehensive Review. Clin. Rev. Allergy Immunol..

[147] Li Y., Wang X., Blau D.M., Caballero M.T., Feikin D.R., Gill C.J., Madhi S.A., Omer S.B., Simões E.A.F., Campbell H. (2022). Global, Regional, and National Disease Burden Estimates of Acute Lower Respiratory Infections Due to Respiratory Syncytial Virus in Children Younger than 5 Years in 2019: A Systematic Analysis. Lancet.

[148] Tovo P.-A., Garazzino S., Savino F., Daprà V., Pruccoli G., Dini M., Filisetti G., Funiciello E., Galliano I., Bergallo M. (2023). Expressions of Type I and III Interferons, Endogenous Retroviruses, TRIM28, and SETDB1 in Children with Respiratory Syncytial Virus Bronchiolitis. Curr. Issues Mol. Biol..

[149] Gonzalez-Cao M., Iduma P., Karachaliou N., Santarpia M., Blanco J., Rosell R. (2016). Human Endogenous Retroviruses and Cancer. Cancer Biol. Med..

[150] Xue B., Sechi L.A., Kelvin D.J. (2020). Human Endogenous Retrovirus K (HML-2) in Health and Disease. Front. Microbiol..

[151] Rangel S.C., da Silva M.D., da Silva A.L., Dos Santos J.D.M.B., Neves L.M., Pedrosa A., Rodrigues F.M., Trettel C.D.S., Furtado G.E., de Barros M.P. (2022). Human Endogenous Retroviruses and the Inflammatory Response: A Vicious Circle Associated with Health and Illness. Front. Immunol..

[152] Gagliardi A., Porter V.L., Zong Z., Bowlby R., Titmuss E., Namirembe C., Griner N.B., Petrello H., Bowen J., Chan S.K. (2020). Analysis of Ugandan Cervical Carcinomas Identifies Human Papillomavirus Clade–Specific Epigenome and Transcriptome Landscapes. Nat. Genet..

[153] Alldredge J., Kumar V., Nguyen J., Sanders B.E., Gomez K., Jayachandran K., Zhang J., Schwarz J., Rahmatpanah F. (2023). Endogenous Retrovirus RNA Expression Differences between Race, Stage and HPV Status Offer Improved Prognostication among Women with Cervical Cancer. Int. J. Mol. Sci..

[154] Soleimani-Jelodar R., Arashkia A., Shoja Z., Akhavan S., Yarandi F., Sharifian K., Farahmand M., Nili F., Jalilvand S. (2024). The Expression Analysis of Human Endogenous Retrovirus-K Env, Np9, and Rec Transcripts in Cervical Cancer. J. Méd. Virol..

[155] Ogata T., Okui N., Sakuma R., Kobayashi N., Kitamura Y. (1999). Integrase of Human Endogenous Retrovirus K-10 Supports the Replication of Replication-Incompetent Int- Human Immunodeficiency Virus Type 1 Mutant. Jpn. J. Infect. Dis..

