# Supplementary material for: Transactivation of Human Endogenous Retroviruses by Viruses
Source: Viruses. 2024 Oct 22;16(11):1649. doi: 10.3390/v16111649 (PMC11599155; doi:10.3390/v16111649)
Supplement: Supplementary file 1 [file viruses-16-01649-s001.zip › viruses-3180257-supplementary.pdf]

Supplementary Table S1. List of differentially expressed HERVs.

Virus	Evidence of HERV transactivation	Cell line/samples used	Methods	Reference
HIV	↑ HERV-K (HML-2) RNA	HIV+ patient PBMCs	RT-qPCR	Bhardwaj et al., 2014
	↑ HERV-K pol RNA	HIV+ patient plasma	RT-qPCR	Contreras-Galindo et al., 2006
	↑ HERV-K (HML2) RNA and proteins	Primary PBMCs and lymphocytes, H9, U87MG	RT-qPCR, flow cytometry	Contreras-Galindo et al., 2007a
	↑ HERV-K (HML2) RNA and proteins	HIV+ plasma	RT-PCR, Western blot	Contreras-Galindo et al., 2012
	↑ HERV-K RNA	HIV+ PBMCs	RT-PCR	Garrison et al., 2007
	↑ HERV-K (HML-2) gag RNA and protein	Jurkat	RT-PCR, Western blot	Gonzalez-Hernandez et al., 2012
	↑ 26 proviruses, including K108 and K115	Primary lymphocytes	RNAseq, RT-qPCR	Gonzalez-Hernandez et al., 2014
	↑ HERV-K102 pol	HIV+ plasma	RT-qPCR	Laderoute et al., 2007
	↑ or no change HERV-K (HML-2) gag RNA, HERV-K RNA	HIV+ blood, MT2, H9 cells	RT-qPCR	Li et al., 2021
	↑ HERV-K env RNA and protein	Lymphocytes	RT-qPCR, IF	Michaud et al., 2014
	↑ HRES-1/Rab4 protein	HeLa, Jurkat, H9, PBMCs, CD4+ T cells	Western blot	Nagy et al., 2006
	↑ ERV9 solo LTR (LTR12C)	Primary T cells	RNAseq	Srinivasachar Badarinarayan et al., 2020
	↑ or ↓ MSRV, Syncytin1 RNA and protein	U87MG, PHFA	RT-qPCR, Western blot	Uleri et al., 2014
	↑ HERV-E, HERV-T, HERV-W, ERV-9, HERV-K (HML-2, -3, -4, -10)	LC5, LC5-RIC	RT-qPCR	Vincendeau et al., 2015
	↑ or unchanged HERV-K (HML-2) RNA	CD4+ T cells	RT-qPCR, RNAseq	Young et al., 2021
HTLV	↑ HERV-W8, -W18, -H, -K, -E LTR RNA	Jurkat	RT-PCR	Toufaily et al., 2011
HSV-1	↑ RT activity, MSRV virions particles	LM7 cells	Product-enhanced reverse transcriptase (PERT) assay; Electron microsopy	Perron H et al. (1993)
	↑ RT activity	MS patient PBMCs	PERT assay	Brudek T et al. (2007)
	↑ HERV-K RNA	Tera2 cells	Dot blot analysis, electromobility shift assays (EMSA)	Kwun H J et al. (2002)
	↑ HERV-W RNA	HeLa cells	RT-PCR, EMSA	Lee W J et al. (2003)
	↑ HERV-W env and gag	SK-N-MC	RT-qPCR	Nellaker C et al. (2006)
	↑ HERV-W env and gag	IMR-32, HBMEC, primary human cerebral endothelial cells	RT-qPCR	Ruprecht K et al. (2006)
VZV	↑ RT activity	PBMCs (clinical samples)	PERT assay	Brudek et al. (2007)
HCMV	↑ HERV-K RNA	Human embryonic lung fibroblasts	RT-PCR	Nelson P N et al. (1999)
	↑ HERV-K and HERV-W pol RNA	HCMV+ Patient sera	RT-qPCR	Bergallo M et al. (2015)
	↑ RT activity, HERV RNA	GliNS1, G179NS, U373, LNCaP and PC3 cells	Retroviral microarray RT-qPCR	Assinger A et al. (2013)
	↑ HERV-K102 pol RNA	HCMV+ patient plasma	RT-qPCR	Laderoute et al. (2007)
HHV-6	↑ MSRV env, gag RNA and protein	HSB-2, U87, primary cord blood mononuclear cells	RT-qPCR, flow cytometry, IF	Charvet B et al. (2018)
	↑ HERV-K18 env RNA	HSB2-ML and HSB2	RT-qPCR	Tai A et al. 2009
	↑ HERV-K18 env RNA	PBMCs	RT-qPCR	Turcanova V et al. 2009
EBV	↑ MSRV env, Syn1, HERV-W env	MS and healthy donor PBMCs	RT-PCR, flow cytometry	Mameli et al., 2012
	↑ MSRV env RNA	EBV+ and EBV- PBMCs	RT-PCR	Mameli et al., 2013
	↑ HERV-W env RNA	MS PBMCs	RT-qPCR	Perez-Perez et al., 2022
	↑ HERV-K (HML-2) gag RNA	HFLS	RT-qPCR	Freimanis et al., 2010
	↑ HERV-K (HML-2) gag RNA	LCLs	RNAseq, RT-qPCR	Wieland et al., 2022
	↑ HERV-K18 env RNA	Primary B cells	RT-PCR	Sutkowski et al., 2001
	↑ HERV-K18 env RNA	LCLs	RT-PCR	Sutkowski et al., 2004
	↑ HERV-K18 env RNA	Human tonsil cells and mouse splenocytes	RT-qPCR	Hsiao et al., 2006
KSHV	↑ HERV-K env RNA	HUVEC, HIV+ PBMCs	RT-qPCR	Dai et al., 2018
IAV	↑ HERV-W env and gag RNA	CCF-STTG, U937, 293F	RT-qPCR	Nellaker C et al. 2006
	↑ HERV-W gag, ERVWE1	CCF-STTG, primary human fibroblasts	RT-qPCR	Li F et al. (2014)
	↑ ERVV-1 and ERVV-2 RNA	A549, MRC-5	RT-qPCR	Schmidt et al. (2019)
	↑ ERV3 loci	A549	RNAseq	Liu H et al. (2022)
SARS-CoV-2	↑ HERV-W env and protein	SARS-CoV-2+ patient PBMCs	RT-qPCR, flow cytometry	Balestrieri E et al. (2021)
	↑ HERV-W env and protein	Patient PBMCs and plasma	RT-qPCR, flow cytometry, IF	Charvet B et al. (2023)
	↑ HERV-W env and HERV-K env	Nasal swabs, FaDu cells	RT-qPCR	Petrone V et al. (2023)
	↑ HERV-W env	Patient plasma	Simple Western technology	Gimenez-Orenga K et al. (2022)
	↑ HERV-K pol, HERV-H pol	Patient whole blood	RT-qPCR	Tovo et al. (2021)
	↓ HERV-W env, MSRV env	Patient whole blood	RT-qPCR	Tovo et al. (2021)
	↑ HERV-K gag	Primary monocytes	RT-qPCR	Temerozo JR et al. 2022
	↑ Anti-HRV-K env IgG	Patient plasma	Suspension multiplex immunoassay analysis	Apostolou E et al 2022
	↑ HERV-H, HERV-E and HERV-3	A549 and Calu-3	Locus-specific transcriptome analysis	Marston JL et al. 2021
	↑ Various HERVs	Bronchoalveolar lavage fluid	Locus-specific transcriptome analysis	Marston JL et al. 2021
	↑ LTR69	Calu-3	RT-qPCR	Arora A et al. 2023
	↓ HERV-H, HERV-W, HERV-K RNA	Patient PBMCs	RNAseq	Grandi N et al. 2023
RSV	↓ HERV-H pol, HERV-K pol, HERV-W pol, Syn1 env, Syn2 env RNA	Patient whole blood	RT-qPCR	Tovo P-A et al, 2023
CVB-4	↑ HERV-W env RNA and protein	Human pancreatic cells and monocytes	RT-qPCR, Western blot	Dechaumes et al., 2020
DENV	↑ & ↓ Various HERV RNA	A549	RNAseq	Wang M et al. (2020)
Arboviruses	↑ & ↓ HERV RNA	Human primary astrocytes	RNAseq	De Castro F et al. (2022)
HCV	↑ HERVK-102 pol RNA	Patient plasma	RT-qPCR	Laderoute et al. (2007)
	↑ HERV-K (HML2) RNA	Patient PBMCs	RT-qPCR	Weber M et al. (2021)
	↑ HERV-K, HERV-H, HERV-W pol RNA	Leukocytes	RT-qPCR	Tovo P-A et al. (2020)
HBV	↑ HERV-K102 pol RNA	Patient plasma	RT-qPCR	Laderoute et al. (2007)
	↑ HERV-W env RNA and protein	HepG2	RT-qPCR, Western blot	Liu C et al. (2017)
